# Hitting Hotspots: Spatial Targeting of Malaria for Control and Elimination

**DOI:** 10.1371/journal.pmed.1001165

**Published:** 2012-01-31

**Authors:** Teun Bousema, Jamie T. Griffin, Robert W. Sauerwein, David L. Smith, Thomas S. Churcher, Willem Takken, Azra Ghani, Chris Drakeley, Roly Gosling

**Affiliations:** 1 Department of Immunity and Infection, London School of Hygiene & Tropical Medicine, London, United Kingdom; 2 Department of Medical Microbiology, Radboud University Nijmegen Medical Centre, Nijmegen, The Netherlands; 3 Department of Epidemiology, Biostatistics, and HTA, Radboud University Nijmegen Medical Centre, Nijmegen, The Netherlands; 4 Department of Infectious Disease Epidemiology, MRC Centre for Outbreak Analysis and Modeling, Imperial College London, London, United Kingdom; 5 Johns Hopkins Malaria Research Institute and Department of Epidemiology, Johns Hopkins Bloomberg School of Public Health, Baltimore, Maryland, United States of America; 6 Laboratory of Entomology, Wageningen University and Research Centre, Wageningen, The Netherlands; 7 Global Health Group, University of California, San Francisco, San Francisco, California, United States of America

## Abstract

Teun Bousema and colleagues argue that targeting malaria “hotspots” is a highly efficient way to reduce malaria transmission at all levels of transmission intensity.

**Summary Points**

- Heterogeneity is a common facet of infectious diseases, whereby infection and disease are concentrated in a small proportion of individuals.
- In malaria, heterogeneity is manifested as small groups of households, or hotspots, that are at a substantially increased risk of malaria transmission.
- These hotspots exist in all transmission settings but are less easily detected at high transmission intensity.
- Hotspots maintain transmission in low transmission seasons and fuel transmission in the high transmission seasons.
- Targeting hotspots is a highly efficient way to reduce malaria transmission at all levels of transmission intensity.

## Introduction

Current malaria elimination guidelines are based on the concept that malaria transmission becomes heterogeneous in the later phases of malaria elimination [1]. In the pre-elimination and elimination phases, interventions have to be targeted to entire villages or towns with higher malaria incidence until only individual episodes of malaria remain and become the centre of attention [1]. With increasing evidence of clustering of malaria episodes within villages, we argue that there is an intermediate step. Heterogeneity in malaria transmission within villages is present long before areas enter the pre-elimination phase, and identifying and targeting hotspots of malaria transmission should form the cornerstone of both successful malaria control and malaria elimination.

## Heterogeneity, Clustering, Transmission Foci, and Hotspots

Variation in the risk of malaria between villages in endemic regions has long been recognized [2]–[4]. This variation is common for many infectious and parasitic diseases where a small number of human hosts are most frequently or most heavily infected while the majority of a local population carry few or no infections [5]–[8]. In malaria, this is exemplified by a study in Dielmo, Senegal, where children were monitored daily during their first 2 years of life. Some children suffered only one episode of clinical malaria, whilst others suffered up to 20 episodes [9]. In Kenya, researchers noted that malaria exposure could not be homogenous as malaria incidence did not follow a Poisson distribution, a phenomenon they describe as over-dispersion [10]. Over-dispersion is commonly recognized in other infectious diseases, where a small proportion (20%) of the population is responsible for the majority (80%) of transmission, the so-called “20/80 rule” [8],[11]–[13].

Micro-epidemiological variations in malaria exposure are most easily recognized in areas of low or moderate transmission intensity where a considerable proportion of the population may remain malaria free for several years while others experience multiple episodes [8],[11],[14]. In areas exposed to intense malaria transmission, heterogeneity in exposure is also present [15],[16], but may be obscured because the majority of the population experiences at least one infection per year and many infections are carried asymptomatically. At present, the factors underlying the micro-epidemiology of malaria are not fully understood but include variation in distance to the nearest mosquito breeding site, water body or vegetation [14]–[17], household structural features [14]–[17], and both human behavioural [15],[17] and genetic factors [15],[17] that may also result in differential attractiveness to mosquitoes [18]. These factors differ at global and local geographical scales and lead to different and confusing definitions of foci of malaria transmission and hotspots of malaria transmission. Entire countries or islands have been classified as malaria hotspots [19],[20], or the term hotspots of malaria transmission may be reserved for smaller geographical areas [14],[21]–[23], sometimes smaller than 1 km^2^ [22],[23].

## Defining a Hotspot of Malaria Transmission

Two related but distinct geographical units in malaria transmission can be defined: (1) The World Health Organization defines a focus of malaria transmission as a defined and circumscribed locality situated in a currently or former malarious area containing the continuous or intermittent epidemiological factors necessary for malaria transmission. Foci of malaria transmission can be classified as residual active, residual nonactive, cleared up, new potential, new active, endemic, or pseudofoci [1]. In more academic terms, an active focus of malaria transmission is a geographical area that supports malaria transmission, where the local *Anopheles* population sustains the basic reproductive rate (*R*_0_; average number of secondary infections arising in a susceptible population as a result of a single individual with malaria over the course of their malaria infection) at a level above 1 [16]. Its size depends on the mosquito breeding site that forms the centre of the focus and the effective dispersal range of vector mosquitoes, which is several kilometres. The border is the furthest location where malaria is still supported by the breeding site. (2) A hotspot of malaria transmission is defined as a geographical part of a focus of malaria transmission where transmission intensity exceeds the average level. Several hotspots of malaria transmission may be present in a single focus of malaria transmission. Micro-epidemiological conditions for malaria transmission are favourable in a hotspot of malaria transmission, resulting in *R*_0_ estimates that exceed the average for the focus of malaria transmission. The size of a hotspot of malaria transmission is variable but typically <1 km^2^ and smaller than the maximum dispersal range of vector mosquitoes; its borders are defined by the distance from the centre of the hotspot where transmission intensity is no longer (statistically significantly) higher than the average for the focus of malaria transmission [14],[21].

## Why Hotspots Are Important in Malaria Transmission

Heterogeneity in mosquito exposure is key to understanding the differences between foci and hotspots of malaria transmission and their implications for malaria control. Individuals who are bitten most often are most likely to be infected and can amplify transmission by transmitting the malaria parasites to a large number of mosquitoes. Estimates of *R*_0_ are very susceptible to variations in mosquito biting behaviour. *R*_0_ may increase considerably as a consequence of heterogeneity in this behaviour [8],[12]; the susceptibility of *R*_0_ to heterogeneous bitingthis is illustrated by Table 1 where estimates of *R*_0_ increased 1.5- to 4.5-fold as a consequence of introducing heterogeneous biting into a mathematical model of malaria [24] in four villages exposed to moderate transmission intensity in northern Tanzania [14].

**Table 1:** Estimates of the basic reproductive number (*R*_0_) for a given parasite prevalence and heterogeneous mosquito exposure in four villages in northern Tanzania.

Estimate	Manundu	Kilole	Kwagunda	Mkwakwani
Parasite prevalence in 2–9-year-old children	3.3%	8.8%	14.8%	34.0%
Average mosquito exposure in the wet season, mean (standard deviation)	5.1 (22.0)	11.0 (29.6)	19.9 (15.9)	18.9 (19.7)
*R*_0_ assuming homogeneous mosquito exposure	1.4	1.9	2.5	5
*R*_0_ assuming heterogeneous mosquito exposure	5.2	8.7	3.7	11.5

*R*_0_ was calculated by adjusting the mean mosquito exposure to match the equilibrium parasite prevalence for each village, with either homogeneous mosquito exposure or with variation in exposure with the same ratio of standard deviation to mean as observed in that village.

There are two reasons why hotspots are relevant for malaria control [8],[12],[25]. Firstly, if interventions are untargeted, hotspots are likely to be the areas where residual malaria transmission will persist. This hypothesis is supported by observations that hotspots of malaria transmission remained unaltered after overall malaria transmission is reduced [22],[23],[26]. Hotspots of malaria transmission can thereby form a major stumbling block in efforts to eliminate malaria [25].

**Figure 1:**
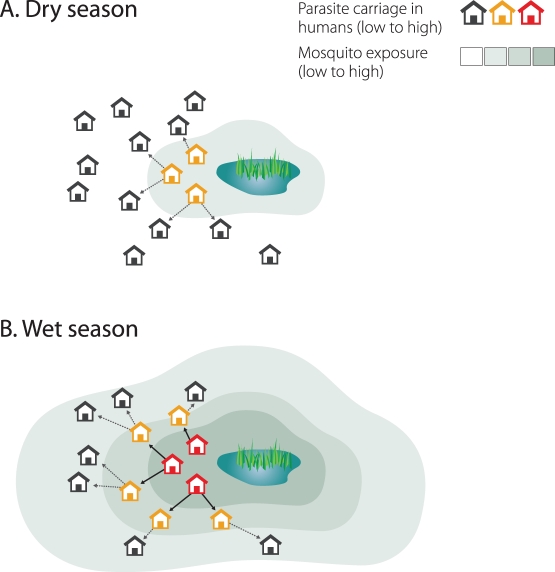
Hotspots of malaria transmission in the dry and wet season. Mosquito exposure and parasite carriage are highly focal in the dry season (A). People living in hotspots are exposed to higher mosquito densities and, because individuals in households belonging to hotspots are more likely to be infected and infectious, mosquitoes are more likely to acquire a malaria infection in these households. In the wet season, as mosquito density and geographic distribution increase, infectious mosquitoes drive infection out into the rest of the village (B).

**Figure 2:**
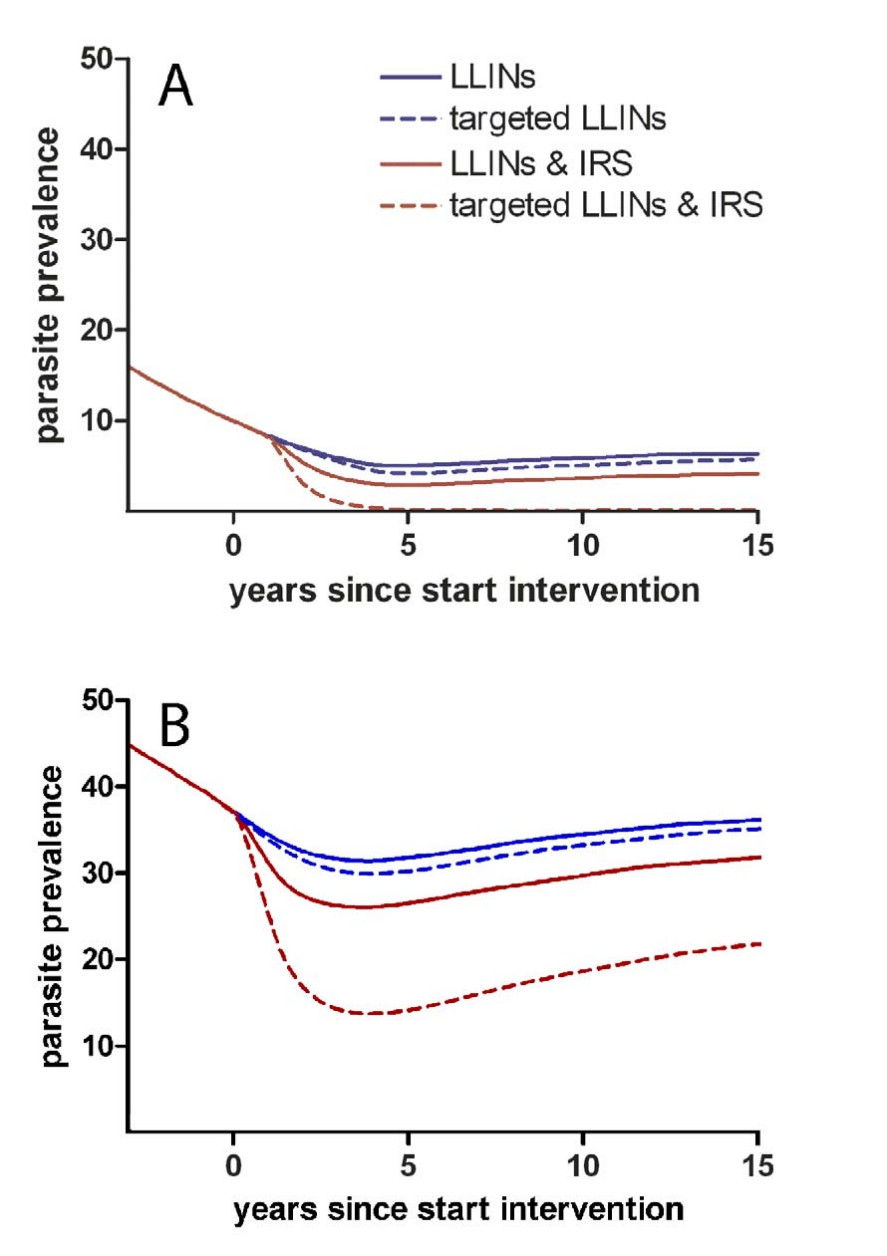
Targeted and untargeted interventions with long-lasting LLINs and IRS in a malaria elimination scenario. The simulations for the low endemic setting with a baseline parasite prevalence of ∼15% in the general population (A) are based on parasite prevalence and mosquito exposure data from Korogwe, northern Tanzania (2008) [14]. Effective coverage with LLINs is scaled up over 6 years to 60% prior to the intervention, creating a starting point for interventions aiming towards malaria elimination [59]. Subsequently, the impact of four intervention strategies is simulated using an individual-based simulation model [24]: (i) increasing LLIN coverage to 80% in a untargeted manner (blue solid line); (ii) increasing LLIN coverage with the same number of LLINs but preferentially targeting hotspots where 90% coverage is reached (dashed blue line); (iii) increasing LLIN coverage to 80% and yearly introducing IRS at 20% coverage in a untargeted manner (red solid line); (iv) a targeted approach using the same resources as the third scenario, reaching 90% effective coverage with LLINs and 90% effective coverage with yearly IRS in hotspots (dashed red line). LLINs were replaced every 4 years. Simulations were repeated for an area of high endemicity with a parasite prevalence of ∼40% in the general population (B).

Secondly, hotspots of malaria transmission are likely to play a catalysing role in areas of stable transmission. Figure 1 shows a schematic of the hotspot theory whereby a few households maintain higher transmission at all time periods. In the dry season the hotspot supports continuing transmission; in the wet season it acts as a source of infection for the rest of the village. This exemplifies the difference between hotspots and foci of malaria transmission: hotspots fuel transmission within transmission foci, whereas foci form independent malarious areas that may contain hotspots. Only the emigration of human parasite carriers or transportation of infectious mosquitoes can result in a spread of parasites beyond the borders of a focus. Interventions targeted at transmission hotspots, but not foci of malaria transmission, therefore have the potential to reduce community-wide malaria transmission. Using the same mathematical model [24] and the same dataset from northern Tanzania [14], we show that targeting hotspots with long-lasting insecticide-treated nets (LLINs) and indoor residual spraying (IRS) could lead to malaria elimination while untargeted interventions with the same resources would lead to more modest reductions in malaria parasite prevalence (Figure 2A). In areas of higher endemicity, targeted interventions alone are unlikely to result in malaria elimination. Nevertheless, also in these settings, targeted interventions have a markedly larger impact compared to untargeted interventions with the same resources (Figure 2B).

## Detecting Malaria Transmission Hotspots

Having argued that hotspots should be targeted, the next obvious question is how can they be identified? Spatial patterns in malaria transmission have been described using (combinations of) micro-epidemiological elevations in malaria incidence [11],[14],[21],[22],[27], asymptomatic parasite carriage [21],[22], reported fever [28], drug use [28], serological responses to malaria-specific antigens [14],[29],[30], mosquito abundance [14],[30], and exposure to infected mosquitoes [14],[30]. Environmental models are very valuable in defining (larger) foci of malaria transmission [31], but currently have limited resolution in identifying small-scale hotspots of malaria transmission within foci of malaria transmission [14],[21].

The most direct evidence of hotspots of malaria transmission is gained by finding an increased exposure to infectious mosquito bites. However, this gold standard measure for defining transmission intensity is extremely laborious and has low sensitivity at low transmission intensity. Furthermore, mosquito sampling strategies for outdoor biting and resting mosquitoes are poorly standardized despite their increasing relevance for transmission [32]. These limitations render an entomological detection of hotspots logistically unattractive.

Clustering of asexual parasite carriage and malaria-specific immune responses currently appear to be the most robust indicators of hotspots of malaria transmission. Incidence of clinical malaria episodes is frequently used as an indicator for increased malaria exposure. However, it should not be used for detecting hotspots unless in an age group defined by low immunity, such as infants or young children, because the higher malaria exposure in hotspots leads to a faster acquisition of immunity against clinical malaria and high density parasitaemia [33]. The likelihood of developing symptoms upon infection may, therefore, be lower in hotspots of malaria transmission. Clustering of asexual parasite carriage forms a more stable indicator of hotspots of malaria transmission than clinical malaria episodes [21], since immunity that prevents malaria infection is acquired later in life, if at all. Antibody responses to malaria-specific antigens can also be used to define small-scale variations in malaria exposure [14],[29],[34]. Because antibody responses are acquired with cumulative exposure and are relatively long-lived, serological markers of malaria exposure are most suitable for detecting stable hotspots of malaria transmission [14] in areas of lower endemicity and can be derived from simple health facility-based surveys [14],[35]. Antibody responses can be analysed as age-dependent sero-conversion rate [14],[36], individual antibody prevalence, or (age-adjusted) individual antibody density [21],[29],[36]. The most suitable approach will depend on the study setting, notably the average level of transmission intensity, and the resolution at which hotspots can (or need to) be detected.

Compared to settings of moderate to low transmission intensity, little research has been done on operational ways to detect hotspots in areas of more intense transmission intensity. Spatial heterogeneity in malaria exposure is common in high endemic settings [15],[26],[36],[37]. Hotspots of malaria transmission, as defined in this manuscript, have been identified by geographical clusters of parasite carriage [26],[36]–[38] and malaria incidence [15]. Serological markers of malaria exposure have been used in high endemic settings [36], but their value for detecting hotspots of malaria transmission against a background of intense transmission intensity remains to be confirmed.

## Practical Arguments That Could Hinder Targeted Control

Three important arguments on hotspots need to be addressed. Firstly, are hotspots stable over time? This is important for practical reasons. Some consistency in the geographical location of hotspots would make implementation of control methods much easier. The predominant observation is that hotspots are remarkably stable even when the intensity of transmission declines [14],[21]–[23],[26],[39],[40]. However, clusters of higher clinical incidence may vary with time [21],[39], especially in settings where outbreaks are related to movement patterns of infected human parasite carriers [22]. In coastal Kenya, evidence was found for the presence of stable and unstable hotspots within the same study population [21].

Secondly, do hotspots seed transmission to the rest of the focus of malaria transmission? The theory behind hotspots fuelling transmission (Figure 1) is supported by several entomological studies that show very focal mosquito exposure in the dry season and more wide-spread mosquito exposure in the wet season while the same households experience the highest relative mosquito [14],[41]–[44] and the highest parasite prevalence in the different seasons [14]. In some areas of low transmission intensity, these persisting hotspots form the only likely source of parasites for seasonal or epidemic increases in malaria transmission in the wider community [22],[40]. Against this are observations that suggest that movement of some vector species is highly localized [45], thereby limiting the spread of malaria from a hotspot to the rest of the village. A study in Tanzania where mosquitoes were captured, marked with fluorescent powder, released, and recaptured observed that 68% of mosquitoes returned to the same household from where they were initially captured [46]. In Papua New Guinea mosquitoes appeared to have a “memorized” home range and limited dispersal range in the focus of malaria transmission they are accustomed to [47]. This nonrandom mixing could have important epidemiological consequences for strategies to control hotspots and would lead to overestimations of impact of hotspot-targeted interventions. In the extreme scenario where mosquito populations do not mix, there would be no community benefit of hotspot targeted interventions. This issue should be addressed in formal evaluations of the community effects of targeted interventions on malaria transmission.

Thirdly, at what geographical resolution can hotspots be detected? The scale at which hotspots are present will greatly influence the feasibility of their identification. Hotspots that are present as geographically clustered groups of households can be more readily identified than smaller hotspots such as individual households. Hotspots may be also more complicated to detect in high endemic settings where the prevalence of malaria parasites and malaria-specific antibodies are high. In these settings, alternative approaches may be needed to determine small-scale variations in transmission intensity. These may include contact tracing of individuals with clinical malaria in the youngest, least immune age groups through health surveillance data or school surveys. More intensive surveillance systems may be capable of combining parasite prevalence and antibody prevalence or sero-conversion rates in young age groups, or examine the number of parasite clones acquired over a certain time period, i.e., the molecular force of infection, once tools are optimised [48].

## When to Target Hotspots of Malaria Transmission

Spatially targeted interventions will not replace the current practice where LLINs and intermittent preventive treatment (IPT) are preferentially provided to young children and pregnant women, groups that are at the highest risk of severe disease. Rather, it will supplement this approach that aims to reduce severe morbidity and mortality with an approach that specifically aims to reduce malaria transmission. Following scaling up in moderate and low transmission settings where malaria transmission is highly heterogeneous, hotspot-targeted interventions form a logistically attractive alternative to untargeted interventions that may need coverage levels nearing 100% to drive transmission lower [8],[12],[14]. To be financially attractive, the costs of detecting hotspots need to be outweighed by the savings made by targeting only a proportion of the total population. For low transmission areas such as those in pre-elimination or elimination phases of malaria control (i.e., malaria incidence below 5 episodes per 1,000 person-years at risk) and in areas that have succeeded in elimination and are preventing re-introduction, the outcome of this equation is very likely to support hotspot-targeted interventions [25]. Hotspot-targeted interventions will also accelerate malaria control in areas of higher endemicity but will require a low-cost and operationally attractive detection system to be financially attractive.

## How to Target Hotspots of Malaria Transmission

The nature of malaria transmission in hotspots, intense mosquito exposure, and high levels of (asymptomatic) parasite carriage in the human population, will require a combination of interventions that target both the human and vector hosts. In addition to scaling up conventional vector control tools such as LLINs and IRS, several less commonly used tools may be particularly suited for hotspots.

### Targeted Vector Control Activities

Conventional vector control activities have largely focused on indoor biting and resting malaria vectors. The role of outdoor biting mosquitoes in malaria transmission is increasingly recognised and their relative importance is increasing with improved coverage of insecticide treated nets and IRS [32],[49]; this poses challenges for vector control that may have to incorporate more laborious components to target outdoor biting vectors. Larviciding of mosquito breeding sites [50] and adult vector control by entomopathogenic fungi [51] both require frequent re-application. Such operational constraints have discouraged widespread adoption, but they may be utilized to great effect as part of a targeting strategy. Similarly, the cost and current efficacy of mosquito traps baited with synthetic human odours [52],[53] make them unlikely candidates to be included in efforts to reduce vector populations at community level although they may hold promise as part of targeted interventions.

### Targeted Interventions to Reduce the Human Infectious Reservoir

The increased parasite biomass in hotspots of malaria transmission in the form of symptomatic and asymptomatic parasite carriers [54] offers the opportunity to reduce malaria transmission by clearing the human parasite reservoir with antimalarial drugs. One possible strategy would be reactive screening and treatment of households and neighbours of individuals who are diagnosed with malaria at health facilities [25]. This approach can be taken a step further by proactive case detection, where people in hotspots are screened for parasitaemia at regular intervals [25]. The most inclusive approach to clear infections in humans, including those that are present at densities below the detection limit of rapid diagnostic tests or microscopy [55] is to give mass drug administration (MDA) where a full therapeutic dose of drugs are administered to a population without prior screening. MDA is a logistically demanding intervention that may need to be repeated several times to maximize its impact [56]. MDA is receiving renewed interest but targeted MDA may be more efficient and high local coverage is more operationally feasible. All three options would ideally employ a drug that actively clears both asexual parasites and gametocytes to rapidly render the treated individual noninfectious [57].

### Targeted Vaccination

Once malaria vaccines become available, they will not only be employed to protect high risk groups against disease and death of malaria but can also play a role in reducing the transmission of malaria. These vaccines that interrupt malaria transmission (VIMT) include vaccines targeting the transmission stages of the parasite and vaccines that reduce the production of gametocytes by targeting pre-erythrocytic and asexual blood stages [58]. Because all age groups contribute to malaria transmission [57], VIMT may need to be administered to all age groups to see an impact. There is currently no infrastructure available for community-wide vaccination campaigns and a targeted approach may therefore be more operationally feasible.

## Conclusions

Malaria hotspots appear to maintain malaria transmission in low transmission seasons and are the driving force for transmission in the high transmission season. Targeting the hotspots would mean the most infected and most diseased households would be prioritized with the added benefits of reducing transmission to the whole community. Identifying the hotspots is possible by mapping asymptomatic carriers or using serological tools. Treating hotspots by ensuring high coverage of interventions for a few households is likely to be easier and much more efficient, and may allow for more complicated interventions than using untargeted approaches. The recent successes of scaling up interventions for impact on malaria have revealed the policy gap of what to do afterwards when coverage is good yet malaria transmission continues. In this paper we have argued that the next evidence-based step is to tackle malaria hotspots. Although knowledge gaps exist, we argue that hotspot-targeted interventions should take place at all transmission levels where resources are sufficient and rapid reductions in malaria transmission will be seen.

## Abbreviations

IRS: indoor residual spraying
LLIN: long-lasting insecticide-treated net
MDA: mass drug administration
